# Polypharmacy, drug-drug interactions, anticholinergic burden and cognitive outcomes: a snapshot from a community-dwelling sample of older men and women in northern Italy

**DOI:** 10.1007/s10433-024-00806-0

**Published:** 2024-03-29

**Authors:** Elena Perdixi, Matteo Cotta Ramusino, Alfredo Costa, Sara Bernini, Silvia Conti, Nithiya Jesuthasan, Marco Severgnini, Federica Prinelli, on behalf of the NutBrain Study Group

**Affiliations:** 1 Department of Neurology, IRCCS Humanitas Clinical and Research Center, Via Alessandro Manzoni, 56, 20089 Rozzano, MI Italy; 2 Neuropsychology Lab/Center for Cognitive Disorders and Dementia IRCCS Mondino Foundation, Via Mondino 2, 27100 Pavia, Italy; 3 Clinical Neuroscience Unit of Dementia, Dementia Research Center, IRCCS Mondino Foundation, Via Mondino 2, 27100 Pavia, Italy; 4 Unit of Behavioral Neurology, IRCCS Mondino Foundation, Via Mondino 2, 27100 Pavia, Italy; 5 Institute of Biomedical Technologies - National Research Council, Via Fratelli Cervi 93, 20054 Segrate, MI Italy

**Keywords:** Older people, Polypharmacy, Drug-drug interactions, Anticholinergic cognitive burden, Cognitive status, Cross-sectional study

## Abstract

Polypharmacy (PP) use is very common in older people and may lead to drug-drug interactions (DDIs) and anticholinergic burden (ACB) that may affect cognitive function. We aimed to determine the occurrence of PP, potential DDIs and ACB and their role in cognitive outcomes in an older population. Cross-sectional data from 636 community-dwelling adults (73.2 ± 6.0 SD, 58.6% women) participating in the NutBrain study (2019–2023) were analyzed. Participants were asked about their medication use, and data on potential DDIs and ACB were extracted. The associations of PP (≥ 5 drugs/day), potential DDIs, and ACB with mild cognitive impairment (MCI) and specific cognitive domains were assessed using logistic regression adjusted for confounders. Sex-stratified analysis was performed. Overall, 27.2% of the participants were exposed to PP, 42.3% to potential DDIs and 19% to cumulative ACB. Women were less exposed to PP and more exposed to ACB than men. In multivariate analysis, the odds of having MCI (24%) were three times higher in those with severe ACB (≥ 3) (OR 3.34, 95%CI 1.35–8.25). ACB was positively associated with poor executive function (OR 4.45, 95%CI 1.72–11.49) and specifically with the Frontal Assessment Battery and neuropsychological tests of phonological and semantic fluency. In sex-stratified analysis, ACB was statistically significantly associated with MCI and executive function in women and with memory in men. PP, potential DDIs and anticholinergics use are very common in community-dwelling older people. ACB exposure is associated with MCI, particularly with poor executive function. Clinicians are encouraged to be vigilant when prescribing anticholinergics.

*Trial registration*: Trial registration number NCT04461951, date of registration July 7, 2020 (retrospectively registered, ClinicalTrials.gov).

**Supplementary Information:**

The online version contains supplementary material available at 10.1007/s10433-024-00806-0.

## Introduction

The use of multiple medications, commonly referred to as polypharmacy (PP), is very common among older adults, especially those with chronic conditions (Masnoon et al. 2017). A prevalence survey in 17 European countries showed that between 26.3% and 39.9% of the population aged ≥ 65 years reported taking at least five different medicines on a typical day, including prescribed drugs, over-the-counter medicines, and dietary supplements (Midão et al. 2018). PP, commonly defined as five or more medications per day (Masnoon et al. 2017), may increase the risk of inappropriate prescribing, poor adherence to treatment, and potential adverse drug events, including potential drug-drug interactions (DDIs), which occur when “the effects of one drug are changed by the presence of another drug” (*MedicinesComplete—Log in*, s.d. Recuperato 10 ottobre 2023, da https://www.medicinescomplete.com/log-in/#/browse/stockley). Prolonged use of PP may lead to a range of multiple adverse effects on physical and functional capacity (Sganga et al. 2014; Fabbietti et al. 2018a, b) and readmission to the hospital (Fabbietti et al. 2018a; b) in older patients. PP has also been found to be associated with poor global cognitive function (Jyrkkä et al. 2011; Cheng et al. 2018) and cognitive ability (Rawle et al. 2018), greater cognitive decline, and dementia or delirium (Oyarzun-Gonzalez et al. 2015). It has also been reported that people with cognitive impairment or dementia take on more drugs than people without cognitive impairment (Lau et al. 2010) due to comorbidity, misinterpretation of adverse drug reactions or newly prescribed medications. This is a risk factor that has been found to be associated with the presence and increased risk of having DDIs (Sönnerstam et al. 2018). In addition, PP may increase the risk of progression from mild cognitive impairment (MCI) to dementia in community-dwelling older adults and the effect appears to be mediated mainly through potential DDIs (Trevisan et al. 2021). However, a clear understanding of the role of PP and DDIs in MCI in free-living older people is far from conclusive.

Many of the medications commonly prescribed to older adults have an anticholinergic (AC) effect, which is the result of a drug interacting with the central nervous system by inhibiting the action of the neurotransmitter acetylcholine that is crucial for learning and memory(López-Álvarez et al. 2019). It is estimated that around 10% of people aged 65 years and older regularly use AC drugs (Chuang et al. 2017), mainly for the treatment of Parkinson's disease, urinary incontinence, Alzheimer’s disease, sleep disorders, depression, and gastrointestinal disorders (O'Donnell et al. 2017). Numerous studies have reported an association between the use of AC drugs and an increased risk of cardiovascular disease and mortality (Myint et al. 2015; Corsonello et al. 2019; D’Alia et al. 2020), as well as potential adverse effects such as cognitive dysfunction, dementia and delirium/confusion (Taylor-Rowan et al. 2021) (Campbell et al. 2009; Pieper et al. 2020). The Anticholinergic Burden (ACB) scale has been developed to identify drugs with AC properties and to guide clinical decisions to reduce and prevent the risk of cognitive adverse events, such as cognitive decline, dementia, and delirium. The ACB scale provides a list of drugs that have AC activity and, because they can cross the blood–brain barrier, may have adverse effects on the central nervous system (Pasina et al. 2019). Several observational studies have investigated the relationship between ACB and cognitive impairment in both clinical (Pasina et al. 2013) and community-dwelling older people, mainly using specific neuropsychological tests without measures of global function or clinical support for neurological assessment (Dos Santos et al. 2022; Han et al. 2008; Koyama et al. 2014; Shah et al. 2013; Uusvaara et al. 2013). A recent systematic review and meta-analysis performed on observational studies, showed that the short- and long-term use of anticholinergics was associated with an increased incidence of dementia and cognitive decline, but no significant association was observed with the incidence of MCI (Pieper et al. 2020).

To the best of our knowledge, data on the occurrence of PP, potential DDIs and ACB in free-living Italian older people, have been less investigated. In addition, their role on cognitive outcomes, using an accurate diagnosis of MCI by combining the neuropsychological and neurological confirmation and examining specific cognitive domains, is rather limited. Finally, although men and women may experience different adverse effects when treated with AC drugs (Trenaman et al. 2021), the sex-specific data on this topic remain scarce. To fill these gaps, the present study aims to provide an overview of the frequency of PP, potential DDIs and AC medication use in a sample of community-dwelling older people, and to examine their independent associations with cognitive impairment and specific cognitive domains separately in men and women.

## Methods

### Study design, setting, and participants

The Nutrition, Gut microbiota, and Brain Aging (NutBrain) project is a population-based cohort study of community-dwelling older adults aged ≥ 65 years living in the Lombardy region (Italy), carried out between October 2019 and January 2023. The procedures of the NutBrain study have been described in detail elsewhere (Prinelli et al. 2020). Briefly, at the time of presentation, individuals were assessed for global cognitive function and specific cognitive domains using a standardized neuropsychological test battery. Socio-demographic and lifestyle characteristics, clinical information and anthropometric measurements were also recorded. All examinations were performed by trained and certified study technicians according to harmonized protocols. A total of 807 participants were recruited; excluding those with missing data on medication use (*n* = 149), those who did not complete the screening data collection (*n* = 4), and those with dementia (*n* = 18) for whom it was not possible to complete the neuropsychological assessment or whose questionnaires responses were not reliable, 636 subjects (*n* = 268 men and *n* = 368 women) were included in the present analysis.

### Data collection

#### Cognitive function

MCI refers to the development of deficits in cognitive abilities, such as memory, attention, language, or executive functions, which are not severe enough to compromise the individual’s functional independence (Albert et al. 2011). In the present analysis MCI was defined as the presence of subjective cognitive complaints and objective cognitive impairment on one or two neuropsychological tests, greater than would be expected for the individual’s age and level of education, without impairment in activities of daily living according to Albert’s criteria (Albert et al. 2011). Functional assessment of activities of daily living was assessed using the Katz Index of Independence in Activities of Daily Living (ADL) scale (Katz 1983) and the Instrumental Activities of Daily Living scale (IADL) (Lawton and Brody 1969). The neuropsychological profile was assessed using a comprehensive battery of neuropsychological tests selected to assess the global cognitive function and different cognitive domains as follows: (i) Memory functions: Free and Cued Selective Reminding Test (FCSRT) (Frasson et al. 2011), Logical Memory Test (Novelli et al. 1986), Rey-Osterrieth Complex Figure Test (ROCF)—delayed recall (Caffarra et al. 2002); (ii) Executive functions: Frontal Assessment Battery (FAB) (Appollonio et al. 2005), Phonemic (Carlesimo et al. 1996) and Semantic Verbal Fluency (Novelli et al. 1986), Trail Making Test part A and B (TMT B) (Giovagnoli et al. 1996); (iii) Language: Picture Naming Test (Sartori and Job 1988); and (iv) Visuospatial abilities: Rey-Osterrieth Complex Figure Test (ROCF)—copy (Caffarra et al. 2002). The results and the participants’ medical records were reviewed by a group of experts, including neurologists and neuropsychologists, to reach a consensus on any controversial cases. All raw neuropsychological test scores were corrected for age and education and compared with those available for the Italian population (Capitani and Laiacona 1997). For each test, the corrected scores were first converted into equivalent scores on a 5-point ordinal scale (Capitani and Laiacona 1988), then the equivalent score were dichotomised into normal (0, scale = 4) and impaired (1, scale 1–4) (Perdixi et al. 2022). We created four cognitive domain scores by summing up all tests contributing to each domain: memory, executive function, language, and visuospatial ability.

#### Use of drugs

Data on the use of each drug were recorded in the database and included the Anatomical Therapeutic Chemical (ATC) classification code, chemical name and brand name. The INTERcheck(^®^) Computerized Prescription Support System (CPSS) (http://www.intercheckweb.it), a database developed by the Mario Negri Institute for Pharmacological Research, Scientific Institute for Research, Hospitalisation and Healthcare (IRCCS), was used to analyze the PP, the potential DDIs and the ACB (estimated using the ACB scale) of the registered drugs. Here a cut-off of ≥ 5 drugs was used to define PP (Masnoon et al. 2017), and the PP score was defined as the total number of drugs taken daily.

The INTERcheck(^®^) system lists information on DDIs based on chemical combinations of drugs. All drug-drug interactions are classified into four different categories according to their clinical significance, as follows: (A) clinically insignificant DDIs; (B) clinical relevance is unknown and/or variable; (C) clinically relevant DDIs that can be managed, for example, by individual dose adjustment; and (D) clinically relevant DDIs that should be avoided. According to previous literature (Ghibelli et al. 2013), each potential DDI was classified as 0 = no drug interaction or minor (A and B), and 1 = at least one from moderate to severe DDI (C and D). The DDI scale indicated the number of drug-drug interactions for each patient. In the ACB scale, drugs were classified according to their AC activity as follows: no activity (score 0), possible activity (score 1), moderate activity (score 2), and severe activity (score 3) (Table 1). The cumulative effect of the ACB score was calculated by summing the ACB score of each drug and then further categorized as 0 (no ACB activity), 1–2 (low to moderate activity), and 3 or more (severe activity).

**Table 1:** Characteristics of the study participants by sex (*n* = 636)

	Total		Men		Women		
	*n* = 636	100%	*n* = 268	42.1%	*n* = 368	57.9%	*p*-value
*Age categories*							
65–69	225	35.6%	84	31.3%	141	38.3%	0.202
70–74	165	25.9%	72	26.9%	93	25.3%	
75–79	131	20.6%	59	22.0%	72	19.6%	
80–84	86	13.5%	36	13.4%	50	13.6%	
85 +	29	4.6%	17	6.3%	12	3.3%	
*Education*							
University	130	20.4%	65	24.3%	65	17.7%	< 0.001
High school	278	43.7%	132	49.3%	146	39.7%	
Middle school	162	25.5%	50	18.7%	112	30.4%	
Primary or less	66	10.4%	21	7.8%	45	12.2%	
*Living condition*							
Not alone	490	77.0%	239	89.2%	251	68.2%	< 0.001
Alone	146	23.0%	29	10.8%	117	31.8%	
*Smoking habit*							
Never	296	46.5%	98	36.6%	198	53.8%	< 0.001
Former	269	42.3%	144	53.7%	125	34.0%	
Current	71	11.2%	26	9.7%	45	12.2%	
*Leisure activities engagement*							
Low	248	39.0%	106	39.6%	142	38.6%	0.858
Moderate	183	28.8%	74	27.6%	109	29.6%	
High	205	32.2%	88	32.8%	117	31.8%	
*Waist circumference (mean. SD)*							
	93.7	12.8	99.8	11.0	89.3	12.4	0.001
*Depressive symptoms (mean, IQR)*							
	7	10	5	6	8	12	< 0.001
*Cardio-metabolic disorders*							
No	124	19.5%	57	21.3%	67	18.2%	0.260
One	192	30.2%	77	28.7%	115	31.3%	
Two	207	32.5%	81	30.2%	126	34.2%	
Three	92	14.5%	40	14.9%	52	14.1%	
Four	21	3.3%	13	4.9%	8	2.2%	
*Mild cognitive impairment, MCI*							
	154	24.2%	68	25.4%	67	18.2%	0.560
*Polypharmacy, PP (≥ 5)*							
	173	27.2%	88	32.8%	85	23.1%	0.006
*Cumulative anticholinergic cognitive burden, ACB*							
No (ACB = 0)	515	81.0%	229	85.4%	286	77.7%	0.016
Low to moderate (1 ≤ ACB ≤ 2)	95	14.9%	34	12.7%	61	16.6%	
Severe (ACB ≥ 3)	26	4.1%	5	1.9%	21	5.7%	
Drug-drug interactions, DDIs (≥ 1)	269	42.3%	119	44.4%	150	40.8%	0.359

*IQR* interquartile range

#### Other assessments

For the present analysis, the potential confounders were selected based on theoretical knowledge from previous epidemiological studies (Han et al. 2008; Koyama et al. 2014; Lau et al. 2010; Shah et al. 2013; Sönnerstam et al. 2018; Trevisan et al. 2021; Uusvaara et al. 2013) and empirical criteria (*p*-value ≤ 0.05 in univariate analysis). We considered sociodemographic information including age (classified as 65–69, 70–74, 75–79, 80–84, and 85 +), sex, education (categorized as university, high school, middle school, primary school or less), and living arrangement (living alone vs not living alone). Health status variables included depressive symptoms as assessed by the 20-item Center for Epidemiologic Studies Depression Scale (CES-D) (Radloff 1977), waist circumference (in centimeters, as a proxy for central obesity), and cardio-metabolic disorders. The cardio-metabolic variable was created by combining self-reported dyslipidemia, hypertension, diabetes, myocardial infarction, stroke, coronary heart disease, peripheral vascular disease, or heart failure. The variable was then categorized as none, one, two, three and four conditions present simultaneously. Lifestyle variables included smoking habits (classified as never and former or current smoker) and the frequency of leisure time activities as assessed by the Cognitive Reserve Index Questionnaire (CRIq) (Nucci et al. 2012). Leisure time activities were first grouped into mental, social, and physical and then categorized into tertiles of engagement as previously described (Perdixi et al. 2022).

### Statistical analysis

Characteristics of the study participants were described using mean (standard deviation—SD) or median (interquartile range, IQR) for continuous variables and frequency (%) for categorical variables. The dependent variables were (i) MCI and (ii) domain-specific cognitive tests (memory, executive function, language, and visuospatial ability). The independent variables were PP, DDIs, and ACB score, considered as both continuous and categorical variables. The association between the dependent and independent variables was examined using binary logistic regression, and odds ratios (OR) with 95% confidence intervals (CI) were estimated. Two sets of models were run, the first including age, sex and education, and the second also including living arrangements, leisure activities, cardio-metabolic disorders, waist circumference, smoking habits and depressive symptoms. A sensitivity analysis was performed after adjustment for the ADL scale to check whether the functional status might affect the results. To account for the potential confounding of co-medications and drug interactions in the assessment of AC effects, we performed a further sensitivity analysis by simultaneously including ACB, PP and potential DDIs in the final model. Sex-stratified analysis was performed. All the analyses were performed with IBM SPSS Statistics for Windows version 25.0 (IBM Corp., Armonk, NY, USA). A two-tailed *p* value ≤ 0.05 was considered statistically significant.

## Results

The 636 participants who met the inclusion criteria were aged 73.17 (SD 6.04) years, 57.9% were women (*n* = 368), 64.1% had completed at least high school, 23% did live alone, 46.5% had never smoked, 32.2% were highly engaged in leisure activities, 30.2% had at least one cardio-metabolic disorders, and 24.2% had MCI. Regarding medications, 27.2% were exposed to PP (continuous score range 0–14), 19% to cumulative ACB (range 0–5) and 42.3% to potential DDIs (range 0–21). Compared to men, women were less educated, more likely to live alone, had never smoked, had a lower waist circumference, had more depressive symptoms, were less exposed to PP but more exposed to severe ACB. There were no differences in age categories, leisure time activities, occurrence of cardio-metabolic disorders, MCI, and exposure to potential DDIs (Table 1).

**Fig. 1:**
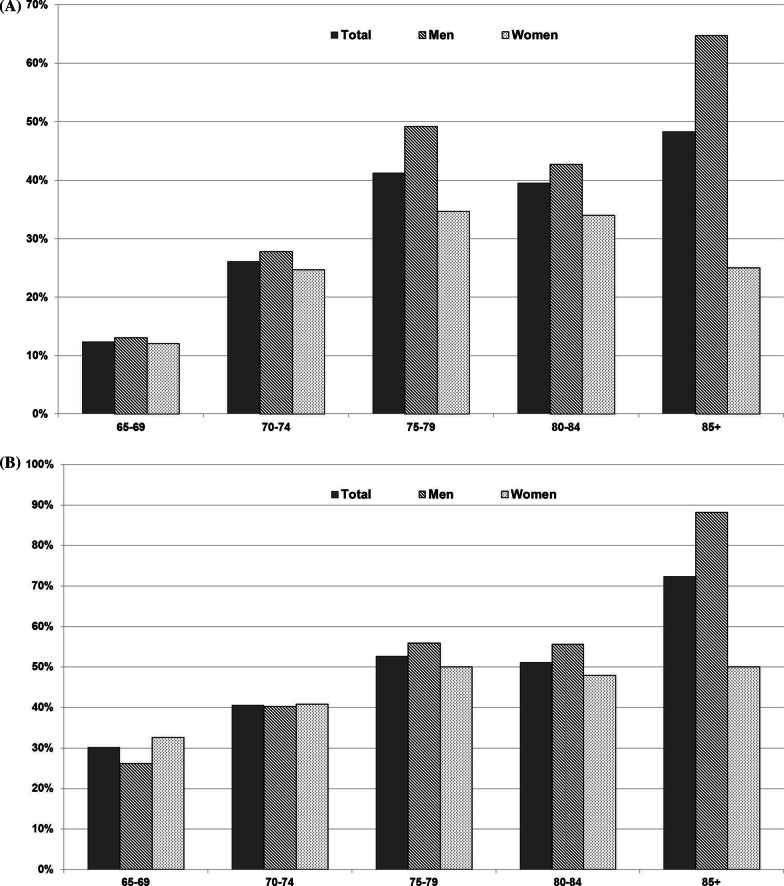
Frequency of polypharmacy (**a**) and drug-drug interactions (**b**) by age classes in men and women

The highest level of PP was found in the 85 + age group (48.3%) (Fig. 1a). There was a sex-related difference in all age groups, with men using more drugs than women. In particular, the difference was significant in the 75–79 age group, where 49.2% of men were exposed to PP compared with 34.7% of women; and in the 85 + age group where 64.7% of men and 25% of women were exposed to PP. We also observed the highest level of potential DDIs in the 85 + age group (72.4%) with a significant sex difference (88.2% of men were exposed to potential DDIs compared to 50% of women) (Fig. 1b).

On average, each participant used 3.2 different therapeutic substances simultaneously (3.5 in men and 3 in women), ranging from 2.2 substances (2 in men and 2.2 in women) in the 65–69 years age group and 4.8 substances (5.5 in men and 3.9 in women) in the 85 + age group (data not shown).

As shown in Supplementary figure 1, the top 5 therapeutic categories (ATC, grouped up to the 2nd level) used by participants included drugs acting on the renin–angiotensin–aldosterone system (39.0%), lipid-modifying substances (34.8%), antithrombotics (30.5%), drugs for acid-related disorders (24.8%) and beta-blockers (22.1%), with women taking fewer antithrombotics, urological agents, drugs for diabetes, cardiac therapy, anti-gout drugs and more psycholeptics, thyroid therapy, and osteoporosis drugs than men.

Supplementary table 1 shows the top 20 most frequent potential DDIs (ATC 5th level), the first 5 being acetylsalicylic acid, levothyroxine sodium, pantoprazole, atorvastatin calcium and bisoprolol.

Supplementary table 2 lists the chemical substances based on their predicted AC effect (clinically relevant to cognition) in the analyzed participants. Drugs with moderate AC effects included amantadine and carbamazepine, whereas drugs with marked AC effects included amitriptyline, clomipramine, paroxetine, olanzapine, quetiapine, solifenacin, and tolterodine.

**Fig. 2:**
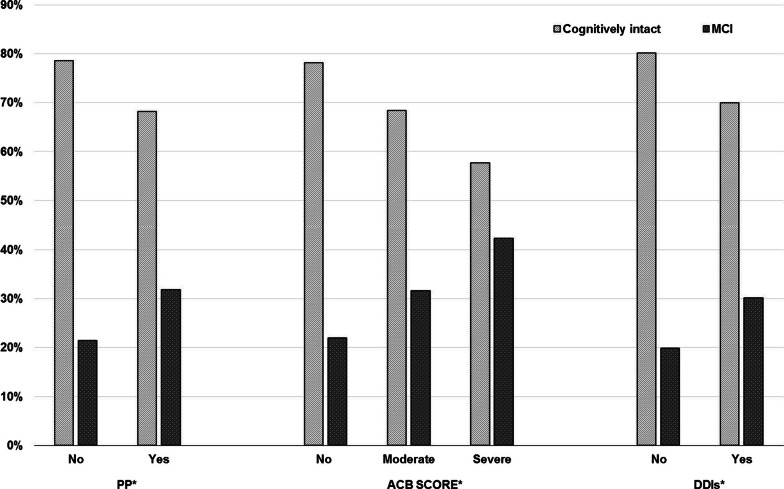
PP: polypharmacy; ACB score: anticholinergic cognitive burden; DDIs: drug-drug interactions. *p-value ≤ 0.05

Compared to those not exposed, the occurrence of MCI was significantly higher in those exposed to PP (21.4% vs 31.8%, *p*-value = 0.006), in those exposed to severe ACB (21.9% vs 42.3%, *p*-value = 0.012), and in those exposed to potential DDIs (19.9% vs 30.1%, *p*-value = 0.030) (Fig. 2).

Table 2 shows the results of the logistic regression model, using the cognitive status as the outcome in the whole sample. We did not find a statistically significant association between cognition, PP and potential DDIs, but we found that those exposed to severe ACB had a high probability of having MCI compared to those not exposed people, considering the model adjusted for age, sex, and education (model 1, OR 3.07, 95%CI 1.27–7.44) and the multivariate model (model 2, OR 3.34, 95%CI 1.35–8.25). Each unit increase in the continuous ACB score was associated with a 1.32 fold increase in the odds of MCI in the multivariate model (OR 1.32, 95%CI 1.04–1.67). Further analysis by including the ADL scale in the model did not alter the associations examined (Additional file 2: Table 3). To exclude that co-medications and drug interactions might confound the effect of anticholinergics on cognitive status, we simultaneously included ACB, PP and potential DDIs in the final model, but the association between ACB and MCI did not change substantially (OR 3.27, 95%CI 1.30–8.24) (data not shown).

**Table 2:** Logistic regression for the association of polypharmacy, ACB score and drug-drug interactions with MCI

	MCI					
	Model 1			Model 2		
	OR	95%CI		OR	95%CI	
*Polypharmacy*						
< 5 drugs/day	Ref	–	–	Ref	–	–
≥ 5 drugs/day	1.02	0.66	1.58	1.03	0.62	1.71
Polypharmacy (one-drug increase)	1.00	0.93	1.09	1.02	0.93	1.13
*ACB score*						
No (ACB = 0)	Ref	–	–	Ref	–	–
Low to moderate (1 ≤ ACB ≤ 2)	1.17	0.69	1.98	1.12	0.65	1.95
Severe (ACB ≥ 3)	3.07*	1.27	7.44	3.34*	1.35	8.25
ACB score (one-unit increase)	1.30*	1.03	1.63	1.32*	1.04	1.67
*Drug-drug interaction*						
< 1	Ref	–	–	Ref	–	–
≥ 1	1.18	0.79	1.77	1.27	0.81	2.00
Drug-drug interactions (one-unit increase)	1.01	0.93	1.10	1.01	0.92	1.11

Model 1 includes age, sex and education

Model 2 includes age, sex, education, living arrangements, leisure time activities, cardio-metabolic disorders, waist circumference, smoking habit, and depressive symptoms

*p-value ≤ 0.05

We also analyzed the association between exposure to ACB and individual cognitive domains (Table 3) controlling for confounders and we found that people exposed to severe ACB had a higher adjusted OR of having impaired cognitive executive function (OR 4.45, 95%CI 1.72–11.49). For each unit increase, the odds increased by 1.44 times (OR 1.44, 95%CI 1.12–1.19). No associations were observed with memory, language and visuospatial ability. Looking at the tests assessing executive function, severe ACB was statistically significantly associated with FAB (OR 4.20, 95%CI 1.54–11.43) and the two tests assessing phonemic (OR 8.26, 95%CI 1.29–53.08) and semantic fluency (17.22, 95%CI 1.78–166.81). For each unit increase in ACB score, the odds of impaired FAB, phonological and semantic fluency increased by 1.38, 1.71 and 2.34 times, respectively (Table 4).

**Table 3:** Logistic regression for the association of polypharmacy, ACB score and drug-drug interactions with impaired cognitive domains

	Memory (*n* = 45)			Executive function (*n* = 100)			Language (*n* = 5)			Visuospatial ability (*n* = 62)		
	OR	95%CI		OR	95%CI		OR	95%CI		OR	95%CI	
*ACB score*												
No (ACB = 0)	Ref	–	–	Ref	–	–	Ref	–	–	Ref	–	–
Low to moderate (1 ≤ ACB ≤ 2)	0.50	0.18	1.38	1.29	0.70	2.37	na			1.08	0.51	2.30
Severe (ACB ≥ 3)	1.19	0.25	5.54	4.45*	1.72	11.49	na			0.93	0.20	4.33
ACB score (one-unit increase)	0.88	0.55	1.42	1.44*	1.12	1.19	na	na	na	1.04	0.73	1.49

Model includes age. sex. education. living arrangements, leisure time activities, cardio-metabolic disorders, waist circumference, smoking habit, and depressive symptoms

*p-value ≤ 0.05

**Table 4:** Logistic regression for the association of ACB score with impaired neuropsychological tests assessing executive function

	FAB (*n* = 74)			TMT (*n* = 28)			Phonological fluency (*n* = 13)			Semantic fluency (*n* = 7)		
	OR	95%CI		OR	95%CI		OR	95%CI		OR	95%CI	
*ACB score*												
No (ACB = 0)	Ref	–	–	Ref	–	–	Ref	–	–	Ref	–	–
Low to moderate (1 ≤ ACB ≤ 2)	1.25	0.64	2.44	1.30	0.46	3.67	3.08	0.75	12.75	0.66	0.05	8.74
Severe (ACB ≥ 3)	4.20*	1.54	11.43	1.04	0.11	10.14	8.26*	1.29	53.08	17.22*	1.78	166.81
ACB score (one unit increase)	1.38*	1.05	1.81	1.15	0.67	1.96	1.71*	1.08	2.69	2.34*	1.26	4.36

Model includes age. sex. education living arrangements, leisure time activities, cardio-metabolic disorders, waist circumference, smoking habit, and depressive symptoms

*p-value ≤ 0.05

Table 5 shows the association between ACB score and cognitive outcomes stratified by sex. We found that women exposed to severe ACB had a statistically significant higher adjusted OR of having MCI (OR 2.90, 95% 1.03–8.15). Severe ACB was significantly associated with impaired memory domain in men (OR 11.12, 95% 1.17–105.38) and with executive function in women (OR 4.71, 95%CI 1.57–14.16).

**Table 5:** Logistic regression for the association of ACB score with MCI and cognitive domains by sex

	Men (*n* = 268)			Women (*n* = 368)		
	OR	95%CI		OR	95%CI	
*MCI*						
*ACB score*						
No (ACB = 0)	Ref	–	–	Ref	–	–
Low to moderate (1 ≤ ACB ≤ 2)	1.07	0.40	2.84	1.11	0.55	2.23
Severe (ACB ≥ 3)	9.14	0.98	84.94	2.90*	1.03	8.15
*Memory*						
*ACB score*						
No (ACB = 0)	Ref	–	–	Ref	–	–
Low to moderate (1 ≤ ACB ≤ 2)	0.98	0.23	4.25	0.26	0.05	1.27
Severe (ACB ≥ 3)	11.12*	1.17	105.38	na		
*Executive function*						
*ACB score*						
No (ACB = 0)	Ref	–	–	Ref	–	–
Low to moderate (1 ≤ ACB ≤ 2)	0.94	0.31	2.80	1.38	0.64	3.01
Severe (ACB ≥ 3)	3.14	0.29	34.30	4.71*	1.57	14.16
*Language*						
*ACB score*						
No (ACB = 0)	Ref			Ref		
Low to moderate (1 ≤ ACB ≤ 2)	na			na		
Severe (ACB ≥ 3)	na			na		
*Visuospatial abilities*						
*ACB score*						
No (ACB = 0)	Ref	–	–	Ref	–	–
Low to moderate (1 ≤ ACB ≤ 2)	1.70	0.40	7.11	0.98	0.40	2.42
Severe (ACB ≥ 3)	7.16	0.37	140.64	0.54	0.07	4.40

Model includes age, education, living arrangements, leisure time activities, cardio-metabolic disorders, waist circumference, smoking habit, and depressive symptoms

*p-value ≤ 0.05

## Discussion

The present study provides a comprehensive picture of medication use in an older sample of men and women living in the community in northern Italy. It also explores the role of PP, potential DDIs and ACB on cognitive outcomes.

Regarding drug use, we found that 27.2% of the study population used at least 5 different therapeutic substances on a daily basis. This percentage increases with age category, ranging from 12.4% in individuals aged 65–69 to 48.3% in those aged 85 + . The prevalence of PP reported in the literature varies widely. A study carried out in the general population of northern Italy using the administrative data reported a prevalence of co-prescription of 31.7% in people aged ≥ 65 years (Valent 2019). A prevalence survey in 17 European countries reported that between 26.3% and 39.9% of the population aged ≥ 65 years reported taking at least five different medicines on a typical day, including prescribed medicines. In Italy, the reported prevalence was 32.9%, increasing with age category from 26.4% in subjects aged 65–74 years to 45.1% in those aged ≥ 85 years, and slightly higher in men (33.2%) than in women (32.5%) (Midão et al. 2018). We found a sex difference in all age groups, with men being more exposed to PP than women (32.8% vs 23.1%), especially in the 70–74 and 85 + age groups. In addition, on average, each participant reported using 3.2 different therapeutic substances simultaneously (range: 2.2–4.8 in the 65–69 and 85 + age groups, respectively). A similar trend was observed in the 2021 report of the Italian Medicines Agency (AIFA) (National Report on Medicines Use in Italy—Year 2021, s.d.). As the report provides a picture of pharmaceutical care in the local and hospital settings rather than in the community, the prevalence of PP was higher than in our study (66.6% vs 27.2%); however, it also showed the same lowest drug use in the 65–69 age group and the highest use in the 85 + age group. In addition, similar to our data, both sexes showed a progressive increase with age in the number of different substances used. Furthermore, in line with the AIFA report (National Report on Medicines Use in Italy—Year 2021, s.d.), the most commonly self-reported drugs in our cohort were antihypertensives, lipid-modifying agents, antithrombotics and drugs for acid-related disorders, with some expected sex differences.

As highlighted by previous studies, PP is associated with a higher risk of potential DDIs. A very recent meta-analysis by Hughes et al. (2023) reported a prevalence of potential DDIs ranging from 0.8 to 90.6% in the older community dwellers. In Italy, in particular, the prevalence reported in three studies of older community dwellers ranged from 19.3% (Burato et al. 2020), 26.4% (Tragni et al. 2013), and 45.0% (Trevisan et al. 2021). In our study, we found that 42.3% were exposed to potential DDIs, with no significant differences between the sexes (44.4% men and 40.8% women) particularly in the 85 + age group. According to the AIFA report (National Report on Medicines Use in Italy—Year 2021, s.d.), we found that the five most common potential DDIs are drugs commonly used in the older population, such as non-steroidal anti-inflammatory drugs (NSAIDs), thyroid hormones, drugs for acid-related disorders, lipid-modifying agents, and beta-blockers.

Regarding the use of AC drugs, in our sample drugs with moderate AC effects include amantadine (antiparkinsonian) and carbamazepine (anticonvulsant or antiepileptic), whereas drugs with marked AC effects that are clinically relevant to cognition include antidepressants (amitriptyline, clomipramine, paroxetine), antipsychotics (olanzapine, quetiapine), and antimuscarinics (solifenacin, tolterodine). This is consistent with the AIFA report (National Report on Medicines Use in Italy—Year 2021, s.d.), which indicates that the therapeutic category of central nervous system drugs appears to have a greater AC effect and includes antiepileptics, antipsychotics, antiparkinsons, and tricyclic antidepressants.

In line with the prevalence in the general population (Sachdev et al. 2015), 24.2% of our sample met the criteria for MCI. Looking at the associations between medication use and MCI, we did not find a statistically significant association with PP and potential DDIs, but we did observe that people exposed to severe ACB had a 3.3 fold increased odds of having MCI. The association was independent of potential confounders, co-medications and potential DDIs. We also found that people exposed to severe ACB were more likely to have impaired cognitive executive function, and we observed greater impairment in skills related to frontal activity measured by the FAB, such as motor and strategic planning and interference control, and in the ability to access the lexicon with both phonemic (phonemic verbal fluency) and semantic (semantic verbal fluency) cues.

Our results are consistent with previous observational evidence showing a consistent association between AC medications use and risk of cognitive decline or dementia in cognitively healthy older adults (Taylor-Rowan et al. 2021) and risk of dementia progression (Trevisan et al. 2021) or poor outcomes, such as mortality, in older adults with pre-existing cognitive problems.

For example, a study of 896 community-dwelling older American Catholic clergy without dementia at baseline, assessed with the MMSE and a neuropsychological battery, reported that people who started AC medication had a steeper annual decline in cognitive function over a median follow-up of 10 years (Shah et al. 2013). Another study of 13,065 community-dwelling Brazilian participants, showed a significant association of ACB with poor memory (CERAD test) and executive function (TMT-B test) in participants aged less than 65 years suggesting that executive function may be altered before other cognitive domains (Dos Santos et al. 2022). Ziad et al. demonstrated a negative cross-sectional association between total cumulative exposure to AC drugs and cognitive performance in 34,267 individuals aged 45–70 years; in particular, the association was moderate for executive function (Digit Symbol Substitution Test, TMT-A and TMT-B) and less pronounced for episodic memory (immediate and delayed free recall) (Ziad et al. 2018). A cross-sectional study conducted in Finland of 400 home-dwelling individuals aged 75–90 years without major clinical dementia but with a history of stable atherosclerotic disease found that drugs with AC properties were associated with lower verbal fluency and naming scores (Uusvaara et al. 2013). Han et al. also showed that cumulative AC exposure from multiple medications over 1-year impaired verbal memory and executive function (using IADL as a proxy) in 544 community-dwelling men aged 65 years and older with diagnosed hypertension (Han et al. 2008). Kyoama et al. found that higher ACB scores were associated with poorer cognitive performance in verbal fluency and immediate and delayed word recall in a 5-year longitudinal follow-up study of 1429 older participants (Koyama et al. 2014). The potential biological basis for the reduced cognitive function associated with the use of medications with moderate or high AC effects was investigated through the functional and structural changes in the human brain, in a longitudinal study of two cohorts of cognitively normal older adults, the Alzheimer’s Disease Neuroimaging Initiative (ADNI) and the Indiana Memory and Aging Study (IMAS). The study showed that AC drugs are associated with poorer cognition (particularly in immediate memory recall and executive function measured by the TMT-B test), brain hypometabolism, whole brain and temporal lobe atrophy, and an increased risk of clinical conversion to cognitive impairment (Risacher et al. 2016). Indeed, the authors speculate that increased brain atrophy and reduced brain function may be related to the central effects of AC drugs on cholinergic pathways in the brain. In a more recent study performed in a population-based cohort of non-demented adults aged from 20 to 80 years, Kilimann et al. reported an inverse association between the ACB and the hippocampal volume (Kilimann et al. 2022).

AC drugs block the binding of the neurotransmitter acetylcholine to cholinergic receptors within the cholinergic system and inhibit its activity at both central and peripheral nervous system synapses. Acetylcholine is a neurotransmitter that plays an important role in many functions of the nervous system and, in the brain, in learning, memory and attention (Klinkenberg et al. 2011; Taylor-Rowan et al. 2022). By interfering with the cholinergic system, together with potential inflammatory, or vascular pathways, AC drugs are thought to affect short and long-term cognitive function, with a greater AC exposure leading to greater impairment (Sanghavi et al. 2022; Singh et al. 2013). In addition, older adults may be more susceptible to the effects of AC medications due to increased permeability of the blood–brain barrier and decreased acetylcholine-induced transmission within the central nervous system (Reiter et al. 2021), as well as age-related changes in drug metabolism and excretion (Trenaman et al. 2021), so  greater caution is required when prescribing AC medications in this population.

Sex-stratified analysis showed that women were more likely to be exposed to severe ACB than their male counterparts (5.7% vs 1.9%), which may be partly because they take more psychoanalytic and psychoanaleptic medications. Women were statistically significantly more likely to have MCI when exposed to severe ACB, and to have poorer executive function. In men, we observed a non-significant increased likelihood, possibly due to the small number of exposed subjects. Severe ACB exposure was associated with impaired memory domain only in men. As previously reported, specific sex-related differences may potentiate the increased exposure to AC drugs by affecting drug metabolism and adsorption, especially in old age. Women have delayed gastric and colonic emptying, higher gastric pH, reduced catechol-O-methyltransferase activity (involved in the metabolism of catecholamine neurotransmitters) and glucuronidation (involved in drug metabolism), and reduced renal clearance, which may affect the absorption of AC drugs (Trenaman et al. 2021).

### Strengths and limitations

Several limitations need to be considered. First, the cross-sectional nature of the study precludes causal inference for the association between ACB score and cognitive outcomes, so a potential reverse causation bias may have influenced our findings. Second, the data on medications use relied on self-report rather than direct ascertainment from medical/prescription records (with detailed information on duration, dose, etc.), which may have led to recall bias and exposure misclassification. However, given the exclusion of people with dementia at baseline, it is unlikely that participants would have reported taking medication that they were not actually taking. Third, drugs use was recorded according to the medication actually taken, without any information about past use (for example, a participant who had taken an AC medication for several years but stopped shortly before the baseline visit would not be recorded as a user). Fourth, although we controlled for several potential confounders, we cannot completely exclude the possibility of residual confounding due to unmeasured factors. Finally, mortality or hospitalization may be outcomes more closely related to polypharmacy or drug-drug interactions than cognitive outcomes analyzed cross-sectionally. Future studies investigating the longitudinal effect of PP and DDIs on the risk of hospitalization, rehospitalization and mortality are warranted. The present study also has several strengths. It provides a picture of drug use in a community-based sample, which is more representative of the general population than samples recruited in clinical settings. The study is also characterized by a detailed assessment of individual’s cognitive function and clinical and behavioral factors, using validated instruments, administered by trained staff, which reduces non-response and recall bias. In addition, the diagnosis of MCI was made by a multidisciplinary consensus expert team based on the uniform application of widely accepted criteria, using a combination of neuropsychological tests and clinical examination, rather than a single screening tool (such as the MMSE) or a single cognitive test, thus avoiding the risk of false negatives. Finally, we examined sex-differences in our analysis, which have been largely unexplored in the literature.

## Conclusions

This study has shown that PP, potential DDIs and anticholinergics use are very common in the community-dwelling older men and women, and that ACB is associated with impaired cognitive function, particularly poor executive function. Further longitudinal studies with detailed assessment of medical/pharmacy records are warranted, as well as studies using both structural and functional brain imaging and biomarker measures to explore the underlying pathophysiological basis of these associations. Our findings have important policy implications and support clinical decision-making about prescribing drugs and AC medications to safely treat people, accounting for their age and sex. Indeed, healthcare professionals need to be aware of the effects of AC drugs and are strongly encouraged to try to reduce an individual’s ACB by de-prescribing commonly used anticholinergics to reduce the risk of developing long-term cognitive problems in cognitively healthy people, or to reduce the rate of cognitive decline and clinical outcomes in those with pre-existing cognitive impairment (Taylor-Rowan et al. 2022).

### Supplementary Information

**Additional file 1**. Supplementary Figure 1: Top ten therapeutic categories (ATC 2nd level) commonly used in men and women. *p-value ≤ 0.05

**Additional file 2.** Supplementary material.

## Abbreviations

PP: Polypharmacy
DDIs: Drug-drug interactions
AChE: Acetylcholinesterase inhibitors
MCI: Mild cognitive impairment
AC: Anticholinergic
ACB: Anticholinergic burden
MMSE: Mini-mental state examination
NutBrain: Nutrition, gut microbiota, and brain aging
ADL: Katz Index of Independence in activities of daily living
IADL: Instrumental activities of daily living scale
FCSRT: Free and cued selective reminding test
ROCF: Rey-Osterrieth complex figure test
FAB: Frontal assessment battery
TMT: Trail making test
ATC: Anatomical therapeutic chemical
CPSS: Computerized prescription support system
IRCCS: Scientific Institute for Research, Hospitalisation and Healthcare
CES-D: Center for epidemiologic studies depression scale
CRIq: Cognitive reserve index questionnaire
SD: Standard deviation
ORs: Odds ratios
CIs: Confidence intervals
AIFA: Italian Medicines Agency
