# Supplementary figures and images for: Polypharmacy, drug-drug interactions, anticholinergic burden and cognitive outcomes: a snapshot from a community-dwelling sample of older men and women in northern Italy
Source: Eur J Ageing. 2024 Mar 29;21(1):11. doi: 10.1007/s10433-024-00806-0 (PMC10980670; doi:10.1007/s10433-024-00806-0)

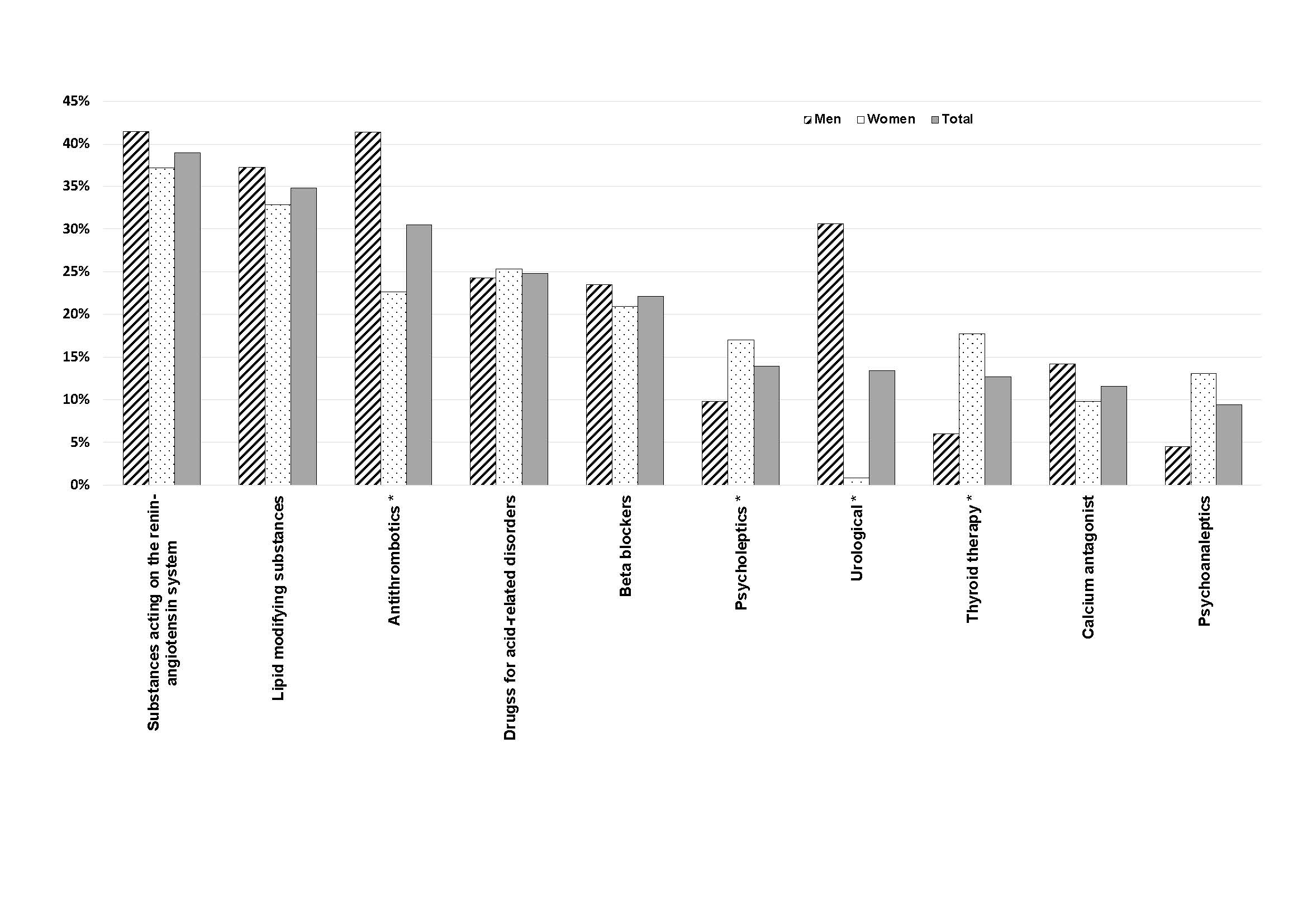

Supplement: Supplementary file 1 — Additional file 1. Supplementary Figure 1: Top ten therapeutic categories (ATC 2nd level) commonly used in men and women. *p-value ≤ 0.05 [file 10433_2024_806_MOESM1_ESM.jpg]
