# Supplementary material for: Polypharmacy, drug-drug interactions, anticholinergic burden and cognitive outcomes: a snapshot from a community-dwelling sample of older men and women in northern Italy
Source: Eur J Ageing. 2024 Mar 29;21(1):11. doi: 10.1007/s10433-024-00806-0 (PMC10980670; doi:10.1007/s10433-024-00806-0)
Supplement: Supplementary file 2 — Additional file 2. Supplementary material. [file 10433_2024_806_MOESM2_ESM.docx]

**Supplementary Table 1. Top twenty most common chemical substances involved in potential DDIs at ATC 5th level in the whole sample.**

| **Chemical substance (ATC 5^th^ level)** | **Frequence (%)** |
| --- | --- |
| Acetylsalicylic acid | 10.9 |
| Levothyroxine sodium | 5.0 |
| Pantoprazole | 4.7 |
| Atorvastatin calcium | 3.9 |
| Bisoprolol | 3.5 |
| Metformin hydrochloride | 3.4 |
| Amlopidine | 3.1 |
| Clopidogrel | 2.9 |
| Esomeprazole | 2.6 |
| Furosemide | 2.5 |
| Omeprazole | 2.3 |
| Ramipril | 2.1 |
| Rosuvastatin | 1.8 |
| Simvastatin | 1.6 |
| Alprazolam | 1.3 |
| Almiloride | 1.3 |
| Atenolol | 1.3 |
| Enalpril | 1.3 |
| Flecainide acetate | 1.3 |
| Lansoprazole | 1.3 |

**Supplementary Table 2. Anticholinergic Cognitive Burden (ACB) score drugs per number of participants**

| **ACB score** | **Chemical substances** |
| --- | --- |
| 1 (n=117)  Drug that exhibits anticholinergic activity in vitro. On a clinical level, cognitive effects are possible, but not very relevant. | Haloperidol, Alprazolam, Atenolol, Metropolol, Atropine, Cetirizine, Colecalciferol, Desloratadine, Digoxin, Diltiazem, Fluvoxamine, Venlafaxine, Furosemide, Isosorbide, Levothyroxine, Nifedipine, Olmesartan, Ranitidine, Oxybutynin, Prednisone, Warfarin. |
| 2 (n=4)  Drug that has moderate anticholinergic effects, which can be clinically relevant on the cognitive sphere. | Amantadine, Carbamazepine. |
| 3 (n=26)  Drug that has marked anticholinergic effects, which are clinically relevant on the cognitive sphere. | Amitriptyline, Clomipramine, Paroxetine, Olanzapine, Quetiapine, Soliphenacin, Tolterodine. |

**Supplementary Table 3. Logistic regression for the association of polypharmacy, ACB score and drug-drug interactions with MCI**

|  | **OR** | **95%CI** | |
| --- | --- | --- | --- |
| **Polypharmacy** |  |  |  |
| **<5 drugs/day** | ref | - | - |
| **≥5 drugs/day** | 1.05 | 0.63 | 1.75 |
| **Polypharmacy (one-drug increase)** | 1.02 | 0.93 | 1.13 |
| **ACB score** |  |  |  |
| **No (ACB=0)** | ref | - | - |
| **Low to moderate (1 < ACB ≤2)** | 1.16 | 0.67 | 2.01 |
| **Severe (ACB ≥ 3)** | 3.35* | 1.36 | 8.25 |
| **ACB score (one-unit increase)** | 1.32* | 1.04 | 1.68 |
| **Drug-drug interaction** |  |  |  |
| **< 1** | ref | - | - |
| **≥ 1** | 1.29 | 0.82 | 2.03 |
| **Drug-drug interactions (one-unit increase)** | 1.01 | 0.91 | 1.11 |

Model includes age, sex, education, living condition, leisure activities, cardiometabolic disorders, waist circumference, smoking habit, depressive symptoms, and Katz Index of Independence in Activities of Daily Living (ADL).

*p-value ≤ 0.05
